# The significance of shooting angle in seal shooting

**DOI:** 10.1017/awf.2024.8

**Published:** 2024-02-06

**Authors:** Kathrine A Ryeng, Stig E Larsen

**Affiliations:** 1 Institute of Marine Research, Fram Centre, PO Box 6606, Stakkevollan, NO-9296 Tromso, Norway; 2 Norwegian University of Life Sciences, Faculty of Veterinary Medicine, Department of Production Animal Clinical Sciences, Ås, Norway

**Keywords:** animal welfare, harp seal, instantaneous death rate, rifle hunt, shooting angle, time to death

## Abstract

The present study aimed to investigate the relationship between shooting angle to the head and animal welfare outcomes in the hunt of young harp seals (*Pagophilus groenlandicus*). The study population consisted of young harp seals belonging to the Greenland Sea harp seal population. A sample of 171, 2–7 weeks old, weaned harp seals of both sexes were included. The study was conducted as an open, randomised parallel group designed trial during the regular hunt. The animals were allocated into four groups, A–D, according to the observed shooting angle to the head, defined as the angle between the direction of the shot and the longitudinal axis of the animal’s head: (A) directly from the front; (B) obliquely from the front; (C) directly from the side; and (D) obliquely or directly from behind. Instantaneous death rate (IDR) and time to death (TTD) were the main variables. The mean IDR differed significantly between groups and was highest in group B (96.8%) and lowest in group C (66.7%). For all groups combined it was 84.2%. The mean TTD for seals not rendered instantaneously unconscious or dead (n = 27) differed significantly between groups and was shortest in group A (16 s) and longest in group C (85 s). However, the number of animals included in the TTD analysis was limited. In conclusion, based on the significantly higher IDR, the shooting angle obliquely from the front is recommended to help achieve the best animal welfare outcomes during the hunt of young harp seals.

## Introduction

Firearms are the most used tool for the killing of seals in the majority of seal hunting regions and countries (The European Food Safety Authority [EFSA] 2007). In the Norwegian commercial harp seal (*Pagophilus groenlandicus*) hunt, young harp seals are today almost exclusively killed with a rifle as the primary weapon. The animals are to be shot when resting on the ice. It is prohibited to shoot seals in the water due to an increased risk of struck-and-lost as compared to seals on ice or land (Sjare & Stenson 2002; Anonymous 2003; North Atlantic Marine Mammal Commission [NAMMCO] 2009). According to the Norwegian seal hunting regulations, the killing should be conducted via a three-step process to minimise the degree of animal suffering. In the first step, the seal is shot to the brain or upper neck, and re-shot, if necessary. The second step requires the shot seal to be approached as soon as possible and struck through the brain with the spike of the *hakapik* (stunning instrument consisting of an iron spike and a hammer mounted on a long wooden pole) as a secondary weapon. If the animal shows any movements upon approach, the blow with the spike should be preceded by a blow to the calvarium with the blunt part of the tool. In the third step, immediately following the second, the insensible animal is bled out through severance of the axillary artery on both sides (Anonymous 2003; Ryeng & Larsen 2021).

An unconscious animal is unable to experience pain (Terlouw *et al.* 2016). Thus, to avoid suffering to the animal, rapid induction of unconsciousness and death is the key issue in any hunt (Aebischer *et al.* 2014; Hampton *et al.* 2015; Ryeng & Larsen 2021). When evaluating animal welfare in hunts that utilise shooting, quantifying the duration of suffering is important to enable comparisons to be made within and between hunts (Hampton *et al.* 2015; Øen 2021). The time to death (TTD) and instantaneous death rate (IDR) are ‘well established’ variables to quantify the animal welfare outcomes in marine mammal hunts (Knudsen 2005; Daoust *et al.* 2013; Hampton *et al.* 2021a; Ryeng & Larsen 2021; Øen 2021), and terrestrial mammal shooting (Cockram *et al.* 2011; Hampton *et al.* 2014, 2015, 2017, 2021b; 2022; Hampton & Forsyth 2016). However, IDR is only really useful for head-shooting practices, i.e. other common terrestrial mammal shooting practices, such as chest shooting, effectively produce IDR values of zero (Stokke *et al.* 2018).

Due to the higher probability of hitting the target, chest shooting is common practice in terrestrial mammal shooting. A bullet impacting major blood vessels, or the heart will cause fatal haemorrhage resulting in hypovolemic shock as the primary cause of death, but there is never an instantaneous loss of consciousness (Newgard 1992; Stokke *et al.* 2018). In seals, by contrast, the target area of the shot is the cranial portion of the central nervous system (CNS) comprising the brain and/or cranial cervical spinal cord to induce instantaneous loss of consciousness and death, thereby preventing the animal from entering the water (Maiden 2009). From an animal welfare point of view, an instantaneous loss of consciousness is desirable (Lewis *et al.* 1997).

The cranium/cranial neck of a seal is a small target area, and only small movements of the head may result in non-fatal hits. The accuracy of the shot is influenced by many factors, particularly the shooter’s skills, shooting distance, stability of the shooting platform and the bullet’s ballistics (Aebischer *et al.* 2014; Massaro 2017). The exterior ballistics, i.e. the path of the bullet through the air, is affected by wind, gravity and friction, and thus by shooting distance and the conditions under which the hunt is conducted (Massaro 2017). In the pack ice hunt, there may be changing weather conditions, bobbing movements of the vessel as well as the ice floe upon which the seal(s) are resting and by movements of the animal itself (Ryeng & Larsen 2021). In such circumstances, the shooter’s experience and judgement are of utmost importance. In the Norwegian hunt for young harp seals, the shooting distance averages around 35 m (Ryeng & Larsen 2021). Such a short shooting distance increases the probability of hitting the target, since it reduces the bullet’s time in flight and makes it less affected by the outer forces (Aebischer *et al.* 2014; Massaro 2017; Hampton *et al.* 2021a).

In wound ballistics, the study of the bullet’s action in tissue, the location of a bullet entrance wound, the path of the bullet, the kinetic energy imparted by the bullet, and deformation/fragmentation of the bullet are the most important factors in causing significant injury or death (Maiden 2009; Kazim *et al.* 2011; Kneubuehl *et al.* 2011). Hence, in terrestrial game hunting, animal orientation (Aebischer *et al.* 2014) or the angle at which the animal is standing in relation to the hunter is of critical importance for the efficacy of the shot, and therefore an important part of hunters’ training and ‘Best Practice’ guidelines in many countries (e.g. Hunter-ed 2004; Best Practice Guide for Scotland 2023). The broadside shot is recommended as it presents the largest target area involving the heart and other vital structures in the thorax. As the shot angle becomes narrower from the broadside position the target area for vital organs becomes significantly smaller. Also, in modern whaling, the angle of the shot relative to the animal’s long axis, was found to significantly influence TTD (NAMMCO 2015; Øen 2021). In sealing, a clear relationship between IDR and the shooting angle to the head, defined as the angle between the direction of the shot and the longitudinal axis of the animal’s head, was detected (Ryeng & Larsen 2021).

The aim of the present study was to investigate the relationship between shooting angle and the animal welfare outcomes in the hunt of young harp seals.

## Materials and methods

### Study animals

The study population consisted of young harp seals belonging to the Greenland Sea harp seal population. A sample of 171, 2–7 weeks old, weaned harp seals of both sexes were included in the study. One hundred and fifty of the animals were identical to those included in Ryeng and Larsen (2021). Additionally, another 21 animals, shot with the default Varmint bullet, were included in the present study. These animals were not included in the former study due to non-compliance with the predetermined bullet type randomisation table (Ryeng & Larsen 2021). The animals were killed as part of a planned hunt which is legal, but strictly regulated in Norway (Anonymous 2003) and would have been killed irrespective of whether they were subjects in the current study.

### Study design

The study reported in Ryeng and Larsen (2021) was conducted as an open, controlled, and randomised parallel group designed trial related to bullet type during the regular hunt of young harp seals. Consequently, this design also applies to the present study.

Based on a video of each animal during shooting and examination of the head post mortem, the animals were retrospectively allocated into four shooting angle groups (A–D). The shooting angle to the head was defined as the angle between the direction of the shot and the longitudinal axis of the animal’s head: (A) 0° = directly from the front; (B) 0° < *x* < 90° = obliquely from the front; (C) 90° = directly from the side; and (D) 90° < *x* ≤ 180° = obliquely or directly from behind (Ryeng & Larsen 2021).

### Study procedure

The study procedure was identical to that described in Ryeng and Larsen (2021). All animals were shot by the same shooter from a stand at the bow of the vessel equipped with a wooden platform as a gun rest. Animals were included in the study in the same order as they were hunted and numbered consecutively. Each animal was observed by a veterinarian prior to and during shooting. A video of each animal was made from close to the shooter’s stand, starting prior to shooting and ending after the animal was bled.

As soon as possible after shooting, the animal’s state of consciousness was clinically assessed by a veterinarian or under veterinary supervision. The following signs of an effective stun/kill were used: immediate collapse; total body relaxation; absence of the corneal and righting reflexes; apnoea; no recovery of rhythmic respiration or any breathing movements of the chest or nostrils; and presence of uncontrolled tonic or clonic spasms, referred to as post mortem reflex movements. The degree of damage to the skull was investigated visually and via palpation.

Animals assessed as being unconscious or dead after being shot, and re-shot if necessary (step one), were immediately bled (step three), omitting the use of the secondary weapon (step two). Animals showing any voluntary movements of the head, body, flippers or any other sign of consciousness, including fear-induced paralysis (Lydersen & Kovacs 1995), were immediately stunned with the secondary weapon and bled in line with the regulations (step two and three). The interventions of assessing the animals’ state of consciousness after shooting and potentially omitting the second step of the prescribed three-step killing process were subject to ethical review by the Norwegian Animal Research Authority and approved under permit number 2014/6264.

The Norwegian Directorate of Fisheries granted dispensation to omit the second step in the prescribed three-step killing process according to the study protocol. No animal was struck-and-lost. A post mortem examination of each head was performed aboard the ship on the same day.

### Study variables

The TTD and IDR were the main variables in the study. The TTD was defined as the time (s) until irreversible unconsciousness or death occurred in animals that survived the first shot that hit the animal. The IDR was defined as the proportion of animals rendered instantaneously irreversibly unconscious or dead from the first shot. Consequently, IDR was not included in TTD. The TTD and IDR recordings were made retrospectively from the video of each animal, based on the animal’s reaction to the shot and findings from the clinical and post mortem examinations.

The secondary variables were as follows: bullet impact site; entrance wound location; shooting distance; the number of shots and missed shots per animal; time between the first and last shot to hit the animal; immediate collapse; degree of body relaxation, post mortem reflex movements (PMRM) variables; visible bleeding; bleeding intensity; time to onset of voluntary movements; total cranial damage score; and bullet exit wound.

Bullet impact site (defined as the anatomical structures receiving the major damaging effect from the bullet), immediate collapse, the degree of body relaxation, PMRM variables, bleeding variables, and total cranial damage score, are previously described (Ryeng & Larsen 2021). The degree of body relaxation, as observed immediately after shooting, was categorised as ‘total relaxation’ with no movements of head, body or flippers, as ‘gradual relaxation’ with an initial tone in front or hind flippers that relaxed gradually within a few seconds, and as ‘voluntary movements’ with normal mobility or voluntary movements of the head, body or flippers, or fear-induced paralysis (Lydersen & Kovacs 1995; EFSA 2007). Following total or gradual body relaxation, the PMRM are characterised by clonic contractions, often referred to as ‘swimming reflexes’ (Daoust & Caraguel 2012), or tonic contractions whereby the animal keeps the caudal portion of the body flexed to one side (Ryeng & Larsen 2021).

The bullet entrance wound locations were classified into: Head; cranium, orbit/eye, maxilla/zygomatic arch, mandible/mouth, Neck; cranial or caudal region, which included all structures and tissues in the neck, Body; forelimb/scapula, thoracic back, thorax, abdominal back, abdomen, pelvis, and hindlimb.

All time variables were recorded in seconds. For animals that survived the first shot, the time between the first and last shot to hit the animal and the time from shooting to onset of voluntary movements were recorded. A bullet exit wound was recorded as being either present or absent.

### Statistical analysis

Time-to-event variables are expressed by Kaplan-Meier plot and categorical or discrete distributed variables are expressed in contingency tables. Frequencies are expressed in percent with 95% confidence intervals (CI) constructed using simple binomial sequences (Lee & Wang 2003; Agresti 2013).

Survival Analysis was used for comparison of groups regarding time-to-event variables (Lee & Wang 2003). Contingency Table Analysis was used for categorical or discrete distributed variables corrected for bullet type (Agresti 2013). All tests were performed two-tailed and differences considered significant for *P*-values less or equal to 5%. The data analysis was generated using SAS/STAT software for Windows® (version 9.4, 2016; SAS Institute Inc, Cary, NC, USA).

## Results

For all groups combined, the mean IDR was 84.2% and differed significantly between groups (*P* < 0.01) (Table 1). The highest IDR was detected in group B, followed by groups D and A. The lowest IDR was observed in group C.

**Figure 1.**
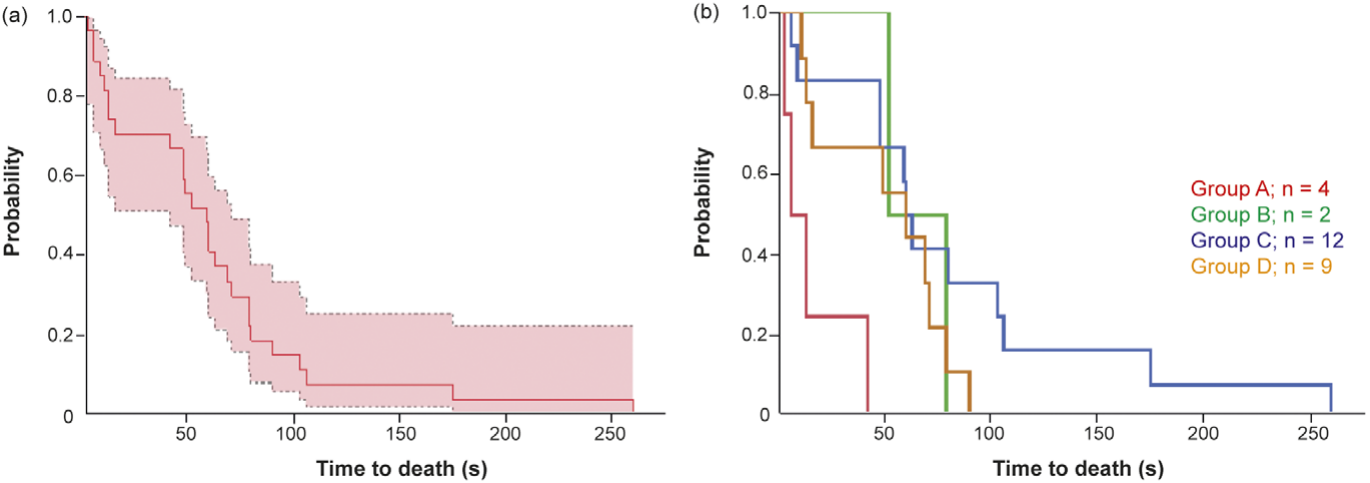
Time to death (s) in 27 young harp seals not rendered instantaneously dead expressed by Kaplan-Meier plot (a) with 95% confidence boundary, and (b) separated into groups (A–D) categorised by shooting angle to the head: (A) directly from the front, (B) obliquely from the front, (C) directly from the side and (D) obliquely or directly from behind.

**Table 1.** Comparison of instantaneous death rate between groups (A–D) of young harp seals categorised by shooting angle to the head. The results are expressed as observed numbers and percent with 95% confidence interval (CI)

Shooting angle groups	Instantaneous death			*P*-value
	Numbers	%	95% CI	
**A** Directly from the front (n = 21)	17	81.0	57.1–94.3	
**B** Obliquely from the front (n = 63)	61	96.8	88.0–99.2	< 0.01
**C** Directly from the side (n = 36)	24	66.7	48.1–81.4	
**D** Obliquely or directly from behind (n = 51)	42	82.4	69.2–91.3	
Total (n = 171)	144	84.2	77.2 –89.4	

Twenty-seven animals were not rendered instantaneously irreversibly unconscious or dead, of which four were in group A, two in group B, 12 in group C, and nine in group D. All the animals in groups A and B were re-shot. In groups C and D, five and seven were re-shot and seven and two were killed with the secondary weapon, respectively.

In all the groups combined the TTD was 62 s (95% CI: 40–84) (Figure 1[a]). The shortest TTD was recorded in group A with a mean of 16 s, followed by group D with 51 s (95% CI: 28–74), group B with 66 s, and group C with 85 s (95% CI: 39–130) (Figure 1[b]). The differences in TTD between groups were significant (*P <* 0.01).

In all groups, bullet impacts to the brain and cranial spinal cord, cranial cervical spine only as well as impacts in close proximity to the cranial CNS, were instantaneously fatal (Table 2). However, one animal in group C did not die instantaneously from an impact to the cranial wall.

**Figure 2.**
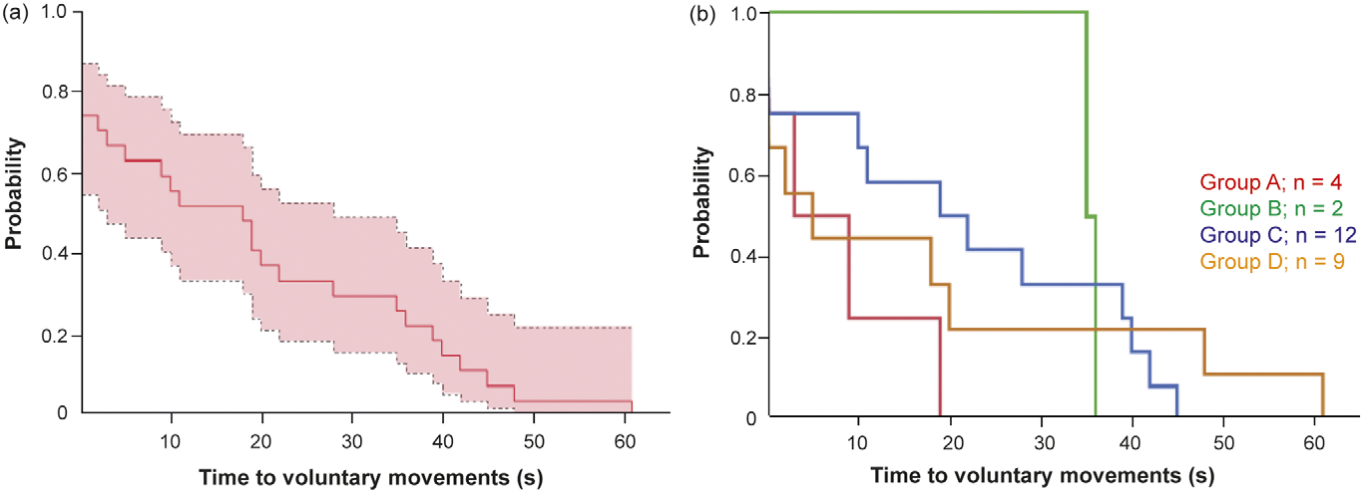
Time from shooting to onset of voluntary movements (s) in 27 young harp seals not rendered instantaneously dead expressed by Kaplan-Meier plot (a) with 95% confidence boundary, and (b) separated into groups (A–D) categorised by shooting angle to the head: (A) directly from the front, (B) obliquely from the front, (C) directly from the side, and (D) obliquely or directly from behind.

**Table 2.** Instantaneous death related to bullet impact site and bullet entrance wound location within groups (A–D) of young harp seals categorised by shooting angle to the head

	Anatomical structures	Shooting angle groups				
		A	B	C	D	Total
Bullet impact site	**Brain and cranial spinal cord**					
	Cranial cavity	11 [11]	44 [44]	20 [20]	33 [33]	130 [129]
	Cranial wall	3 [3]	10 [10]	2 [1]	7 [7]	
	**Cervical spine**					6 [6]
	Cranial spine	1 [1]	3 [3]	2 [2]		
	Caudal spine					
	**Head or neck outside CNS**					29 [9]
	Close proximity to cranial CNS	2 [2]	4 [4]	1 [1]	2 [2]	
	Orbit/eye		1 [0]	3 [0]	1 [0]	
	Maxilla/Mandible			3 [0]	3 [0]	
	Soft tissue neck			1 [0]	1 [0]	
	Skin & blubber	3 [0]		2 [0]	2 [0]	
	**Body** Forelimb or scapula Skin & blubber					6 [0]
		1 [0]	1 [0]	2 [0]	1 [0]	
					1 [0]	
Bullet entrance wound location	**Head**					
	Cranium	4 [4]	32 [32]	19 [18]	19 [19]	74 [73]
	Orbit/eye	3 [3]	9 [8]	4 [1]	1 [0]	17 [12]
	Maxilla / Zygomatic arch	5 [5]	8 [8]	1 [0]	1 [0]	15 [13]
	Mandible / Mouth	3 [3]	8 [8]	2 [0]	2 [0]	15 [11]
	Skin & blubber	1 [0]			1 [0]	2 [0]
	**Neck**					
	Cranial	3 [2]	4 [4]	6 [5]	17 [17]	30 [28]
	Caudal		1 [1]		6 [6]	7 [7]
	Skin & blubber	2 [0]		2 [0]		4 [0]
	**Body**					
	Forelimb or scapula		1 [0]	2 [0]	1 [0]	4 [0]
	Thoracic back				2 [0]	2 [0]
	Skin & blubber				1 [0]	1 [0]
**Total**		21 [17]	63 [61]	36 [24]	51 [42]	171 [144]

A: directly from the front; B: obliquely from the front; C: directly from the side; D: obliquely or directly from behind. Results expressed as observed numbers with the number of instantaneous deaths given in square brackets.

No animal was registered with an impact to the caudal cervical spine only. In group D, impacts to the cervical spine were not registered. A clear pattern was seen between groups regarding the frequency of impacts to non-vital structures within the head or neck outside CNS site, such as orbit/eye, maxilla, mandible, and soft tissue of the neck. The highest frequencies were found in groups C and D.

Except for the above-mentioned animal in group C, bullet entrance in the cranium was instantaneously fatal in all groups (Table 2). A clear pattern was seen between groups regarding IDR and bullet entrance into non-vital structures of the head. In groups A and B, bullet entrance into these structures was instantaneously fatal, but not in groups C and D. Entrance wound locations in the neck, mostly registered in the cranial neck, resulted in instantaneous death in most cases. A higher frequency of both cranial and caudal neck entrance wounds was found in group D. Bullet entrance wounds in the body were mostly located in the forelimb or scapula. However, in one animal in group A registered with an impact to the forelimb/scapula, the bullet entrance wound was found in the cranial neck. In two animals in group D, the entrance wound was located in the thoracic back. In these animals, the bullet impact sites were detected in soft tissue of the neck, and skin and blubber within the head or neck outside CNS site, respectively.

For all groups combined, the mean shooting distance was 36 m (n = 147) (95% CI: 3–38), range: 8–67 m. No significant difference was detected between groups (*P* = 0.19). For animals with TTD = 0 and TTD > 0, the mean shooting distance was 35 m (95% CI: 33–38) (n = 120) and 40 m (95% CI: 36–45) (n = 27), respectively. This difference was significant (*P* = 0.03).

The number of shots and missed shots per animal did not differ significantly between groups (Table 3).

**Table 3.** Comparison of the number of shots and missed shots per animal between groups (A–D) of young harp seals categorised by shooting angle to the head

Variable	Category	Shooting angle groups			
		A	B	C	D
Number of shots per animal	1	17	57	29	39
	2	4	6	5	11
	3	0	0	1	1
	4	0	0	1	0
	*P*-value	0.39			
Number of missed shots per animal	None	19	59	29	40
	1	2	4	5	10
	2	0	0	2	1
	*P*-value	0.12			

A: directly from the front; B: obliquely from the front; C: directly from the side; D: obliquely or directly from behind. Results expressed as observed numbers.

The time interval between the first and last shot to hit the animal, as recorded in 18 animals, was 16 s in group A, 66 s in group B, 40 s (95% CI: 0.4–80) in group C, and 47 s (95% CI: 17–78) in group D. No significant difference between groups B, C and D was detected. The lower time interval in group A was borderline significant, but the number of animals was too low to draw any conclusions.

The degree of body relaxation, occurrence of PMRM, visible bleeding, and the presence of a bullet exit wound differed significantly between groups (Table 4). The largest proportion of animals with total body relaxation was observed in group B with 74.6%, followed by group D with 62.7%, group A with 57.1%, and group C with 55.5%. The occurrence of PMRM was highest in group B with 87.3% and lowest in group C with 55.6%. Visible external bleeding was most frequently observed in group C with 85.7%, followed by group D with 80%, and groups A and B, both with 76.2%. Visible external and internal bleeding with swelling of the head and neck was most frequently observed in group B with 23.8%, followed by group D with 20%, and groups A and C both with 14.3%. The presence of a bullet exit wound was highest in group C with 86.1%, followed by group D with 84.3%, group B with 63.8%, and group A with 28.6%. Borderline significant differences between groups were found regarding immediate collapse and the strength of PMRM, while no significant differences were found for bleeding intensity and the quality of PMRM.

**Table 4.** Comparison of secondary variables between groups (A–D) of young harp seals categorised by shooting angle to the head

Variable	Category	Shooting angle groups				Total
		A	B	C	D	
Immediate collapse	No	2	1	5	3	11
	Yes	19	62	30	48	159
	Total	21	63	35	51	170
	*P*-value	0.09				
Degree of body relaxation	Total relaxation	12	47	20	32	111
	Gradual relaxation	5	14	4	10	33
	Voluntary movements	4	2	12	9	27
	Total	21	63	36	51	171
	*P*-value	0.04				
Occurrence of post mortem reflex movements	None	8	8	16	12	44
	Present	13	55	20	39	127
	Total	21	63	36	51	171
	*P*-value	<0.01				
Quality of post mortem reflex movements	Tonic	6	24	8	19	57
	Clonic	6	30	12	18	66
	Total	12	54	20	37	123
	*P*-value	0.84				
Strength of post mortem reflex movements	Weak	6	17	7	22	52
	Moderate	3	15	9	10	37
	Powerful	3	19	4	5	31
	Total	12	51	20	37	120
	*P*-value	0.11				
Visible bleeding	None	2	0	0	0	2
	External	16	48	30	40	134
	External and internal	3	15	5	10	33
	Total	21	63	35	50	169
	*P*-value	0.02				
Bleeding intensity	Scarce	3	2	5	4	14
	Moderate	3	7	7	5	22
	Excessive	14	54	23	41	132
	Total	20	63	35	50	168
	*P*-value	0.24				
	None	15	21	5	8	49
Bullet exit wound	Present	6	37	31	43	117
	Total	21	58	36	51	166
	*P*-value	< 0.01				

A: directly from the front, B: obliquely from the front, C: directly from the side, and D: obliquely or directly from behind. Results expressed as observed numbers.

For all groups combined, the mean duration of PMRM was 48 s (95% CI: 44–52) and did not differ significantly between groups (*P* = 0.81).

The mean time from shooting to onset of voluntary movements, as recorded in 27 animals, was 19 s (95% CI: 12–26) (Figure 2[a]). No significant difference between groups was detected (*P* = 0.38) (Figure 2[b]). The mean time to onset of voluntary movements was 8 s in group A, 37 s in group B, 21 s (95% CI: 10–32) in C, and 17 s (95% CI: 5–30) in D.

The mean total cranial damage score (n = 151) was 46.3 (95% CI: 43.3–49.4). It showed an increasing trend with the lowest value of 40.9 (95% CI: 30.0–51.8) in group A, followed by group B with 45.6 (95% CI: 40.4–50.8), group C with 46.4 (95% CI: 38.9–53.9) and group D with 49.3 (95% CI: 44.7–54.0). No significant difference was detected between groups (*P* = 0.45), but a borderline significant higher score was detected in group D as compared to group A (*P* = 0.09).

## Discussion

The present study demonstrates that the IDR, and thus the animal welfare outcomes of the hunt, is highly dependent on the shooting angle to the head of young harp seals. While shots obliquely from the front resulted in an IDR close to 100%, it was dramatically reduced for shots directly from the side. Between these two extremes, shots directly from the front or obliquely or directly from behind produced mean IDRs above 80%.

Instantaneous death occurs only if the bullet strikes the brain and/or cranial cervical spinal cord (Maiden 2009). Accordingly, and irrespective of shooting angle, bullet impacts to the cranial CNS, including impacts in close proximity to the cranial CNS were all instantaneously fatal. One animal that was shot directly from the side and classified with an impact to the cranial wall, did not die instantaneously. It was hit tangentially to the calvarium and much of the kinetic energy of the bullet was probably lost outside the head of the animal (Ryeng & Larsen 2021). Impacts in close proximity to the cranial CNS were in most cases characterised by gross subdural and subarachnoid haemorrhages, often massive, particularly on the ventral surfaces of the brain-stem. Such indirect CNS tissue damage occurs through the mechanism of temporary cavitation (Maiden 2009). Vascular injuries in these sensitive and vital parts of the brain have been documented to correlate significantly with mortality (Knudsen & Øen 2003; Øen & Knudsen 2007; Kazim *et al.* 2011; Kneubuehl *et al.* 2011; Ryeng & Larsen 2021).

Bullet entrance wound location and the path of the bullet are important factors in causing significant injury or death (Maiden 2009; Kazim *et al.* 2011; Kneubuehl *et al.* 2011). In the present study, the bullet path, as determined by the shooting angle to the head, was the major factor contributing to the observed differences in IDRs. While bullet entrances into the cranium and most entrances in the neck were instantaneously fatal irrespective of shooting angle, a dependency between IDR and shooting angle was detected for bullet entrances into non-vital structures of the head such as the orbit/eye, maxilla/zygomatic arch, mandible/mouth. While bullet entrance into these structures directly or obliquely from the front resulted in instantaneous death, entrance directly from the side or obliquely from behind resulted in non-fatal wounding. This could be explained by the direction of the bullet path. When placed in these structures directly or obliquely from the front, the bullet will in most cases continue its way through the structure and reach the cranial CNS to cause instantaneous death. If, however, the bullet enters these structures directly from the side or obliquely from behind, the path of the bullet will not strike the cranial CNS but leave its major damaging effect in the non-vital structure and thus only wound the animal.

This also explains the higher frequency of bullet impacts to these structures for shots directly from the side and obliquely from behind, since bullet impact was defined at the anatomical structure receiving the major damaging effects from the bullet.

Twenty-seven animals were not rendered instantaneously unconscious or dead. The TTDs for these animals also indicated a dependency of the shooting angle. The angle directly from the side not only produced the lowest IDR, but also the longest TTDs. Hence, the poor animal welfare outcomes for this shooting angle were shown by both main variables. It should be noted, however, that these results should be interpreted with caution since the number of animals included in the TTD analysis was limited. The longest TTDs were recorded for surviving animals whose signs of consciousness were not observed by the shooter. These were finally killed with the secondary weapon. Most of these animals were shot directly from the side with bullet impacts close to the cranial CNS, such as impact tangential to the cranial wall, impacts to the orbits, throat, maxilla or mandible. The longest TTDs were observed for bullet impact to the orbits, as seen in three animals. In one of these animals (TTD = 260 s), the bullet had passed through both orbits. Such perforating shots perpendicular to the frontal aspects of the cranium are only possible from an angle directly from the side which significantly prolonged the temporary loss of consciousness. In comparison, impacts to the orbit obliquely from the front and obliquely from behind, as detected in one animal in each group, both resulted in a TTD of 79 s.

The shortest TTD was detected for shots directly from the front. Here, three of the four animals were hit in the skin and blubber of the head or neck. Such superficial hits cause immediate or early signs of consciousness, and the animals are rapidly re-shot. Hence, the recorded TTD became shorter.

The mean TTDs observed for shots obliquely from the front and obliquely or directly from behind (groups B and D) were intermediate to those observed for shots directly from the front and directly from the side (groups A and C). In group D, impacts to the maxilla or mandible resulted in the longest TTDs of up to 90 s, indicating a prolonged temporary loss of consciousness as these structures are impacted obliquely from behind.

In all groups, impacts to the body were almost exclusively located in the forelimb or scapula, producing TTDs between 13 and 71 s. Impacts to the forelimb may be explained by the way the seal body is oriented relative to the shooter rather than the shooting angle to the head. In four of the five cases where the impact was on the forelimb or scapula, the animals were lying with the hindlimbs directed towards the shooter. In such situations, the likelihood of bullet impact to the forelimb or scapula is rather high from any shooting angle to the head if the bullet hits too low. For impacts to the forelimb from behind the body, the bullet may continue its way forwards and strike close to the head, which may explain the longest observed TTDs.

It is vital for the animal welfare outcomes of probably any seal hunt that the shooter is aware of the risk of wounding the animal if aiming for the head directly from the side or obliquely from behind. Although impacts to the cranial CNS from these angles are equally efficient, hits slightly rostral to the cranium may result in some of the worst welfare outcomes, namely snout shots. Particularly for longer shooting distances or during sub-optimal shooting conditions, these angles should be avoided, or the hunt should be halted until the shooter can be sure that the bullet will enter the cranium behind the eyes. The present study demonstrates that the most efficient shooting angles to the head are obliquely from the front followed by directly from the front and directly from behind. This is opposite to the recommended broadside position or shot angle of 90° relative to the longitudinal axis of the animal’s body in terrestrial mammal shooting where the thorax is the target area (e.g. Hunter-ed 2004; Best Practice Guide for Scotland 2023).

The mean shooting distance did not differ significantly between shooting angle groups. However, it was significantly shorter for animals that died instantaneously as compared to those that did not. This underlines the importance of shooting distance for the accuracy of the shot, even for the short distances operated here (Aebischer *et al.* 2014).

The time to onset of voluntary movements was the shortest time interval as compared to the time from first to last shot to hit the animal and TTD. This could be explained by the fact that voluntary movements must occur prior to re-shooting. Also, it is easier to detect the onset of such movements from the video than in real time. Hence, the mean time from first to last shot to hit the animal, as detected by the shooter, was longer, and equivalent to TTD, for the 18 animals that were re-shot. No significant difference was detected between groups regarding these two variables, indicating that the time to regain consciousness was similar, regardless of shooting angle.

The degree of body relaxation and occurrence of PMRM differed significantly between groups. These variables are closely related to the degree of damage to the cranial CNS (Ryeng & Larsen 2021). Total body relaxation is associated with complete destruction of the brain and brain-stem and was mostly represented by cases with severe primary brain damage (EFSA 2007). Animals with ‘gradual relaxation’ had less destructive but lethal primary brain damage (Finnie 2016), while ‘voluntary movements’ were entirely represented by the 27 animals not rendered instantaneously irreversibly unconscious or dead. The occurrence of PMRM, which indicates a successful killing in seals, likely results from the loss of higher motor control following acute trauma to the head or neck (Daoust & Caraguel 2012; Verhoeven *et al.* 2015; Ryeng & Larsen 2021). The largest proportions of animals with total body relaxation as well as occurrence of PMRM were observed in group B, followed by groups D, A and C, the same order as decreasing IDRs between groups. The proportions of animals with ‘voluntary movement’ as a sign of consciousness, followed the opposite order with the largest proportion observed in group C, followed by groups A, D and B. These patterns confirm the close relationship between these two variables and IDR.

Visible bleeding also differed significantly between shooting angle groups and correlated closely to the degree of damage to structures of the head and cranial neck, including blood vessels. While external bleeding alone was most frequently observed in group C, followed by groups D, A and B, external bleeding combined with internal bleeding seen as swelling of the head and cranial neck region was most frequently observed in group B, followed by groups D, A and C. The latter phenomenon developed within seconds of impact and was mainly associated with bursting injuries with total disintegration of cranium and brain architecture, being consistent with the fact that most impacts to the cranial cavity and cranial wall were seen in group B, followed by groups D, C and A. Such bursting injuries may be explained by temporary cavity formation within the skull from high-velocity bullets (DiMaio 2016). It seems likely that the swelling was caused by the pumping of arterial blood from disrupted arteries into the large permanent wound cavity of the head covered by the skin (Ryeng & Larsen 2021).

The presence of a bullet exit wound also differed significantly between groups and was highest in group C, followed by groups D, B, and A. Hence, the probability of a perforating shot was highest for shots fired to the head directly from the side and lowest for shots fired directly from the front. This was expected, since the shortest and the longest path through the head of a seal would be right from the side and right from the front, respectively. Consequently, shots directly from the side should be performed with caution not only because of the risk of wounding the animal but also for the risk of accidental injuries to neighbouring seals caused by perforating shots (Ryeng & Larsen 2021).

Borderline significant differences between groups were found regarding immediate collapse. Following the same order as the IDR, the highest proportion of animals showing immediate collapse, an indicator of the potential loss of consciousness (Terlouw *et al.* 2016; American Veterinary Medical Association [AVMA] 2020), was observed in group B, followed by groups D, A and C. However, in all groups, the proportion of animals displaying immediate collapse was higher than the IDR, demonstrating that the transfer of kinetic energy from the bullet was sufficient to induce a temporary loss of consciousness even in animals that survived the first shot (Kneubuehl *et al.* 2011; Finnie 2016). Immediate collapse should therefore not be relied upon as the only evidence for the efficacy of the shot.

Unlike the occurrence of PMRM, the quality, strength and duration of PMRM did not differ significantly between the shooting angle groups. Hence, provided sufficient damage to the cranial CNS has occurred to induce loss of higher motor control and thus PMRM, the quality, strength and duration of these movements seemed to be the same, regardless of shooting angle. However, significant differences were detected for these variables when comparing the effects of the fragmenting Varmint bullet to the mushrooming Bonded bullet (Ryeng & Larsen 2021).

The mean total cranial damage score showed an increasing trend from group A through groups B and C to D, reflecting a more damaging effect to the cranium as the shooting angle to the head increases. This was expected since impacts directly to the cranium, and thus more kinetic energy transfer to the cranium, are more likely to occur as the shooting angle increases (DiMaio 2016).

## Animal welfare implications and conclusion

The present study demonstrates that the shooting angle obliquely from the front is recommended to achieve the best animal welfare outcomes of the hunt of young harp seals. Shots directly from the side should be performed with caution or avoided.
